# BAASNet: boundary-aware deep learning for accurate polyp segmentation in colonoscopy

**DOI:** 10.1038/s41598-026-65397-5

**Published:** 2026-08-05

**Authors:** Khola Naseem, Nabeel Khalid, Andreas Dengel, Sheraz Ahmed

**Affiliations:** 1 RPTU University Kaiserslautern-Landau, Kaiserslautern, 67663 Germany; 2 German Research Center for Artificial Intelligence (DFKI) GmbH, Kaiserslautern, 67663 Germany

**Keywords:** Colonoscopy, Polyps, Deep learning, Boundary-aware segmentation, Attention mechanisms, Encoder–decoder architecture, Cancer, Computational biology and bioinformatics, Gastroenterology, Mathematics and computing

## Abstract

Colorectal polyps are primarily detected through colonoscopy, which plays a central role in early cancer prevention. Precise polyp segmentation supports treatment planning and diagnostic accuracy by providing masks that encode clinically relevant structures. Recent advancements in deep learning have led to several polyp segmentation models. However, performance remains hindered by challenges such as image noise, complex textures, indistinct boundaries, and diverse polyp morphologies. The high cost and time burden of manual annotation underscore the need for automated segmentation systems. To overcome these limitations, BAASNet, a Boundary-Aware Attention-Based Segmentation framework, is introduced for polyp segmentation. A boundary-aware loss function is integrated to improve performance, particularly in delineating polyp edges. The method is evaluated on nine publicly available datasets spanning five imaging modalities, including two center-wise polyp detection benchmarks, demonstrating strong generalization capability. On PolypDB, the model attains a mean Dice similarity coefficient (mDSC) of at least *89.60%* across all five modalities. Across all evaluated benchmarks, the proposed model achieves an average absolute improvement of approximately *3.3%* in Dice. Gains vary by dataset, ranging from approximately *0.7%* to *4.7%* relative improvement over the best previous results. These results demonstrate BAASNet’s potential for robust, real-time clinical deployment in automated colonoscopy workflows.

## Introduction

Colorectal cancer (CRC), a malignancy affecting the large intestine, ranks among the most prevalent cancers globally^1^. The five-year survival rate varies considerably based on the cancer’s stage and location. CRC is the second leading cause of cancer-related deaths in both men and women. The incidence of CRC is rising rapidly, accounting for approximately *10%* of global cancer cases and around *9.4%* of cancer-related mortality. Notably, the incidence rates among individuals under 50 years of age are increasing at an annual rate of 1–*2%*.

CRC typically originates as small benign polyps that may progress to malignancy over time. Therefore, early detection and removal are essential. Colonoscopy is a standard procedure for both the detection and prevention of CRC, offering detailed visualization of polyp location and appearance. However, the procedure is not infallible; back-to-back colonoscopies can miss between *15%* and *30%* of polyps, particularly those smaller than 10 mm^2^. In such cases, automated systems may improve detection and segmentation accuracy. Advancements in deep learning have led to the development of numerous models aimed at improving polyp segmentation. Over the past decade, the growing adoption of convolutional neural networks (CNNs) has led encoder–decoder architectures to emerge as a dominant baseline for medical image segmentation. These models extract local semantic information through layered convolution operations. However, a key limitation of CNNs lies in their inability to capture long-range dependencies due to the inherently local nature of convolutional operations. This results in a trade-off between computational efficiency and the capacity to capture global contextual information.

To overcome the limitations of traditional CNNs, recent polyp segmentation studies have explored enhanced CNN-based models, Transformer networks, and hybrid CNN–Transformer architectures^3,4^. Transformer-based methods have become a strong direction in medical image segmentation because self-attention enables effective modeling of global contextual relationships, which is particularly useful for distinguishing polyps from visually similar surrounding mucosa^5–7^. However, global context alone is not sufficient for accurate polyp segmentation, as many polyps have blurred boundaries, low contrast, irregular shapes, and weak separation from adjacent tissue. For this reason, recent studies increasingly emphasize edge guidance and boundary refinement to improve contour localization^8,9^. In this context, task-specific boundary-aware loss functions are important because they complement region-based losses such as Dice and cross-entropy by directly encouraging better alignment between predicted and ground-truth contours^10^. Despite these advances, performance inconsistencies across datasets, imaging modalities, and evaluation protocols still limit real-world applicability^11,12^. Moreover, many existing models are trained mainly on white-light imaging (WLI), which limits their generalization to other modalities such as Flexible Spectral Imaging Color Enhancement (FICE), Linked Color Imaging (LCI), Blue Laser Imaging (BLI), and Narrow Band Imaging (NBI). These challenges highlight the need for a generalized boundary-aware segmentation framework that can operate robustly across diverse clinical imaging conditions.

To address these limitations, BAASNet is introduced as a boundary-aware attention network designed for robust and generalized polyp segmentation across diverse datasets and imaging modalities. The proposed architecture integrates a Boundary-Aware Attention (BAA) module to enhance focus on edge pixels, an Atrous Spatial Pyramid Pooling (ASPP) module for multi-scale feature extraction, and a boundary-aware loss function.

- BAASNet combines boundary-aware attention, a boundary-aware loss, and ASPP in a unified encoder–decoder architecture. The Boundary-Aware Attention module fuses edge probability maps into self-attention via learnable modulation and residual fusion, achieving sharper boundary delineation without sacrificing contextual semantics.
- A boundary-aware loss function is introduced, integrating Binary Cross-Entropy (BCE), Dice Loss, and an edge-based BCE term to improve boundary delineation accuracy.
- BAASNet is extensively evaluated on nine public datasets spanning five imaging modalities, including data from nine centers reported in two multi-center datasets, and achieves strong performance across cross-dataset experiments, with up to *90.75%* mIoU on PolypDB. Specifically, the proposed method yields an average mDice improvement of approximately *3.3%* over the best previous results across the evaluated benchmarks.
- Two critical aspects of the proposed approach are emphasized: (1) cross-modality generalization, demonstrated by consistent performance across five endoscopic imaging modalities, and (2) label efficiency, showing that BAASNet maintains high segmentation accuracy with substantially reduced annotation effort. These characteristics are particularly important for future clinical translation, where data heterogeneity and annotation cost are major constraints.

## Dataset

The proposed method is evaluated on nine publicly available polyp segmentation datasets: Kvasir-SEG^13^, PolypDB^14^, EndoTect^15^, CVC-ColonDB^16^, ETIS^17^, CVC-300^18^, CVC-ClinicDB^1^, PolypGen^19–21^ and CVC-EndoSceneStill^18^. These datasets vary in imaging modality, polyp size, and structural complexity, providing a comprehensive benchmark for evaluating segmentation performance. These datasets encompass five distinct imaging modalities: WLI, FICE, LCI, BLI, and NBI, ensuring a diverse test set for evaluating modality-generalized segmentation. The details of each dataset are as follows:

Kvasir-SEG consists of 1000 polyp images, each accompanied by a manually annotated ground truth mask. Of these, 900 images are used for training and 100 for testing. Image resolutions vary between *332× 487* and *1920× 1072* pixels. PolypDB is a recently released dataset containing 3, 934 polyp images with corresponding ground truth masks. The images were obtained using five imaging modalities: FICE, BLI, LCI, WLI, and NBI. The dataset is split into *80%* for training, *10%* for validation, and *10%* for testing.

CVC-EndoSceneStill is a merged dataset combining CVC-ColonDB and CVC-ClinicDB, consisting of 912 manually segmented White Light Images (WLI). The default split provided with the dataset is used for training, validation, and testing.

The PolypGen dataset^20^ contains 1537 polyp images collected from colonoscopy examinations at six centers, representing diverse patient cohorts and populations. The image resolution varies from *384× 288* to *1920× 1080* pixels. Following the recommended evaluation protocol^20^, 1449 images from centers C1–C5 are used for training, while 88 images from the held-out center C6 are reserved for out-of-sample testing.

EndoTect Dataset includes a total of 1200 images. Specifically, 1000 images from Kvasir-SEG are used for training, while an additional 200 images from EndoTect are used for testing. The dataset follows a predefined train-test split to ensure consistency.

To further assess generalization, the trained model was evaluated on unseen images from the test sets of CVC-ColonDB, ETIS, CVC-300, and CVC-ClinicDB, using the same test splits reported in^22^. In addition, results on the PolypDB multi-center dataset are reported; this dataset includes images acquired at three clinical centers (BKAI, Karolinska, and Simula). Detailed dataset statistics for all nine datasets are provided in (Supplementary Table 15 and Supplementary Table 16).

## Results

This section presents a comprehensive evaluation of the proposed BAASNet in comparison with existing state-of-the-art (SOTA) approaches. Specifically, we benchmark BAASNet against recent attention-based networks (e.g., CaraNet, DDANet, PolypSeg+) and Transformer-based approaches (e.g., SSFormer-L, DuAT, FocusNet). As shown in Tables 1 and 2, BAASNet consistently outperforms both categories, achieving higher mDSC and mIoU scores across multiple datasets. These results demonstrate that our boundary-aware attention design provides state-of-the-art accuracy and strong generalization, even when compared with the latest transformer-based architectures. In addition, we conduct detailed ablation studies to evaluate the contribution of each component of BAASNet. Comprehensive performance results across all datasets are presented as follows.

### Results on the PolypDB dataset

The results across all imaging modalities BLI, FICE, LCI, NBI, and WLI are shown in Table 1.

**Table 1:** Segmentation performance comparison on the PolypDB dataset. The best values are in **bold**.

Methods	PolypDB(BLI)		PolypDB(FICE)		PolypDB(LCI)		PolypDB(NBI)		PolypDB(WLI)	
	mIoU	mDSC	mIoU	mDSC	mIoU	mDSC	mIoU	mDSC	mIoU	mDSC
CaraNet^23^	58.53	72.37	56.94	62.96	76.00	85.76	72.49	80.90	85.82	91.28
FocusNet^5^	74.60	82.47	82.53	88.46	86.13	92.04	74.67	82.09	88.62	**93.42**
DuAT^6^	67.37	80.48	55.89	67.46	85.51	91.94	74.94	82.60	88.21	92.94
SSFormer-L^7^	67.50	78.48	76.07	83.00	85.67	92.07	78.48	84.32	**88.90**	93.32
Proposed Model	**81.79 **± ** 0.99**	**89.60 **± ** 0.67**	**83.89 **± ** 0.50**	**90.54 **± ** 0.33**	**90.75 **± ** 0.41**	**94.94 **± ** 0.22**	**85.94 **± ** 1.70**	**92.05 **± ** 1.27**	**87.41 **± ** 0.62**	**92.33 **± ** 0.43**

For the proposed model, results are reported as mean ± standard deviation over three independent runs. Results for competing methods are reproduced from their respective publications, which do not report standard deviations.

**Fig. 1:**
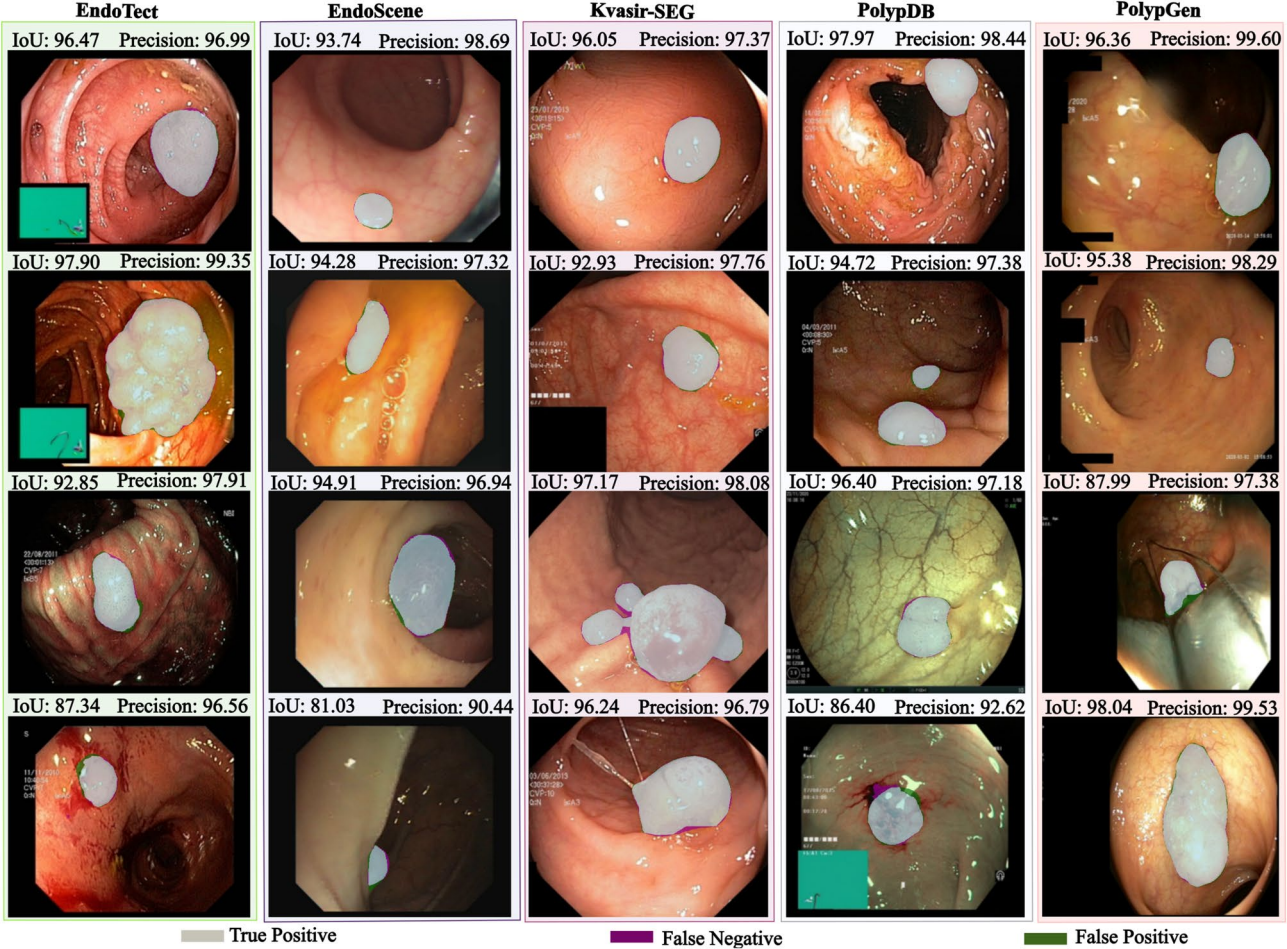
Qualitative analysis of the proposed network’s performance on five different datasets. White regions represent correctly segmented polyps, green regions denote false positives, and purple regions indicate false negatives.

The proposed model achieves consistent gains across most PolypDB modalities. Compared with the strongest baseline for each modality, the proposed model improves mIoU/mDSC by $9.64\%/8.65\%$ on BLI, $1.65\%/2.35\%$ on FICE, $5.36\%/3.12\%$ on LCI, and $9.51\%/9.17\%$ on NBI. On WLI, the proposed model remains competitive, performing within *1.68%* of the best mIoU and within *1.17%* of the best mDSC. Additional evaluation metrics further demonstrate the strong and competitive performance of the proposed method compared with existing approaches. The complete quantitative results are provided in (Supplementary Table 10). In addition, mIoU and mDice results stratified by polyp size are reported in (Supplementary Table 12): small (area < *5%*), medium (*5%* *\ge* area < *15%*), and large (area *\ge* *15%*). Figure 1 shows representative segmentation examples and reports the corresponding IoU and precision values. White regions indicate correctly segmented polyps, green regions denote false positives, and purple regions indicate false negatives.

### Results on the EndoScene dataset

On the EndoScene dataset, the proposed method demonstrates strong performance across all metrics, surpassing existing approaches in terms of both mIoU and mDSC, achieving scores of *81.01* *±* *0.80* and *87.42* *±* *0.59*, respectively, as summarized in Table 2. The proposed method also delivers high performance across other metrics, including precision, recall, accuracy, and F2 score. The detailed quantitative results are provided in (Supplementary Table 11), and the polyp size–wise analysis is reported in (Supplementary Table 12).

**Table 2:** Segmentation performance (mIoU/mDSC) on Kvasir-SEG, EndoTect and EndoScene datasets. “–” denotes missing values; best per-column results are in **bold**.

**Methods**	**Kvasir-SEG**		**EndoTect**		**EndoScene**	
	**mIoU**	**mDSC**	**mIoU**	**mDSC**	**mIoU**	**mDSC**
DDANet^24^	78.00	85.76	70.10	78.74	–	–
NanoNet-A^25^	72.82	82.27	65.00	75.08	–	–
Polypseg+^26^	70.68	78.81	–	–	63.50	71.70
ConvNeXt-Base+MPE^27^	81.63	88.18	79.70	85.65	–	–
Duplex contextual relation^28^	84.44	90.14	–	–	78.86	85.41
**Proposed Model**	**85.62 **±** 0.70**	**90.86 **±** 0.54**	**83.71 **±** 0.36**	**89.67 **±** 0.26**	**81.01 **±** 0.80**	**87.42 **±** 0.59**

For the proposed model, results are reported as mean ± standard deviation over three independent runs. Results for competing methods are reproduced from their respective publications, which do not report standard deviations.

The qualitative results are presented in Figure 1. In the first case, the prediction closely matches the ground truth with minimal boundary errors. The second example shows robust performance under specular highlights and reflections, still capturing the full polyp extent. In the third case, small false negatives occur along low-contrast boundaries, indicating mild under-segmentation. The fourth case is the most challenging, where poor visibility and partial occlusion increase both false negatives and false positives, though the main polyp region is still correctly identified.

### Results on the PolypGen dataset

The results on the PolypGen dataset are shown in Table 3. BAASNet achieves the strongest overall performance on the unseen C6 test set, outperforming existing approaches across most evaluation metrics. It attains the highest IoU (*0.79* *±* *0.25*) and DSC (*0.85* *±* *0.25*), along with the best F2-score (*0.83* *±* *0.25*) and recall (*0.83* *±* *0.26*). BAASNet also remains competitive in Precision (*0.90* *±* *0.24*) and accuracy (0.9845), indicating robust segmentation quality across metrics. In addition, mIoU and mDice results stratified by polyp size are reported in (Supplementary Table 12). Figure 1 visualizes the segmentation results along with the IoU and precision values for each case.

In the first image, the model segments the polyp with near-perfect overlap, and the very high precision (99.60) indicates that almost no background pixels are mistakenly included. In the second image, the method reliably identifies a small, well-circumscribed polyp, with the remaining errors mainly limited to minor boundary under-segmentation. The third image represents a more challenging setting, where specular reflections and low-contrast boundaries introduce ambiguity; nevertheless, the model still captures most of the lesion extent, with only localized false negatives and small false positives near the interface. In the fourth image, despite uneven illumination and strong mucosal texture, the model produces an accurate mask with minimal leakage into the surrounding tissue, consistent with the high IoU (98.04) and precision (99.53).

**Table 3:** Performance comparison on the C6 test set. Values are reported as mean ± standard deviation across test images, following the PolypGen evaluation protocol. Higher is better (*↑*); best score per column is shown in **bold**.

Method	IoU *↑*	DSC *↑*	F2 *↑*	Precision *↑*	Recall *↑*	Acc. *↑*
U-Net^20^	0.55 ± 0.34	0.63 ± 0.36	0.64 ± 0.36	0.76 ± 0.31	0.66 ± 0.37	0.96
PSPNet^20^	0.72 ± 0.27	0.80 ± 0.26	0.79 ± 0.27	0.88 ± 0.20	0.79 ± 0.28	0.98
DeepLabV3+ (ResNet50)^20^	0.75 ± 0.28	0.81 ± 0.27	0.80 ± 0.28	0.92 ± 0.17	0.79 ± 0.29	0.98
DeepLabV3+ (ResNet101)^20^	0.75 ± 0.28	0.82 ± 0.27	0.80 ± 0.27	0.92 ± 0.18	0.81 ± 0.27	0.98
ResNetUNet (ResNet101)^20^	0.74 ± 0.29	0.80 ± 0.28	0.80 ± 0.28	**0.93 ± 0.14**	0.80 ± 0.29	0.98
**BAASNet (Ours)**	**0.79 **±** 0.25**	**0.85 **±** 0.25**	**0.83 **±** 0.25**	0.90 ± 0.24	**0.83 **±** 0.26**	**0.9845**

### Results on the EndoTect dataset

On the EndoTect dataset, the proposed method achieves an mDSC of *89.67 ± 0.26*, as shown in Table 2, and surpasses the strongest existing approaches by *6.4%*, *4.2%*, and *13.1%* in precision, accuracy, and F2-score, respectively. The complete quantitative results are provided in (Supplementary Table 11). Polyp size–wise mIoU and mDice results are reported in (Supplementary Table 12). Figure 1 shows that the proposed method produces high-quality segmentations with strong overlap with the ground truth across varied appearances and illumination. The predictions remain stable under texture variations and non-uniform lighting, with only localized differences near ambiguous boundaries in the most challenging low-contrast case.

### Results on the Kvasir-SEG dataset

Table 2 reports the Kvasir-SEG results, where the proposed method achieves an mDSC of *90.86 ± 0.54* and outperforms the strongest baselines by 2.2, 6.1, 8.2, and 2.83 percentage points in recall, precision, F2-score, and accuracy, respectively. Complete results are provided in (Supplementary Table 11). As shown in Figure 1, the first example illustrates accurate segmentation of a small, well-defined polyp with a smooth contour and minimal background inclusion (IoU = 96.05). The second example demonstrates stable performance on heterogeneous mucosa, capturing the lesion with only minor boundary deviations (IoU = 92.93). The third example highlights robustness on a large polyp with specular reflections and complex appearance, where the predicted mask covers the main lesion while exhibiting small localized discrepancies near the margins (IoU = 97.17). The fourth example further shows reliable delineation for a polyp adjacent to folds, preserving the overall shape with slight contour variation (IoU = 96.24).

## Discussion

**Fig. 2:**
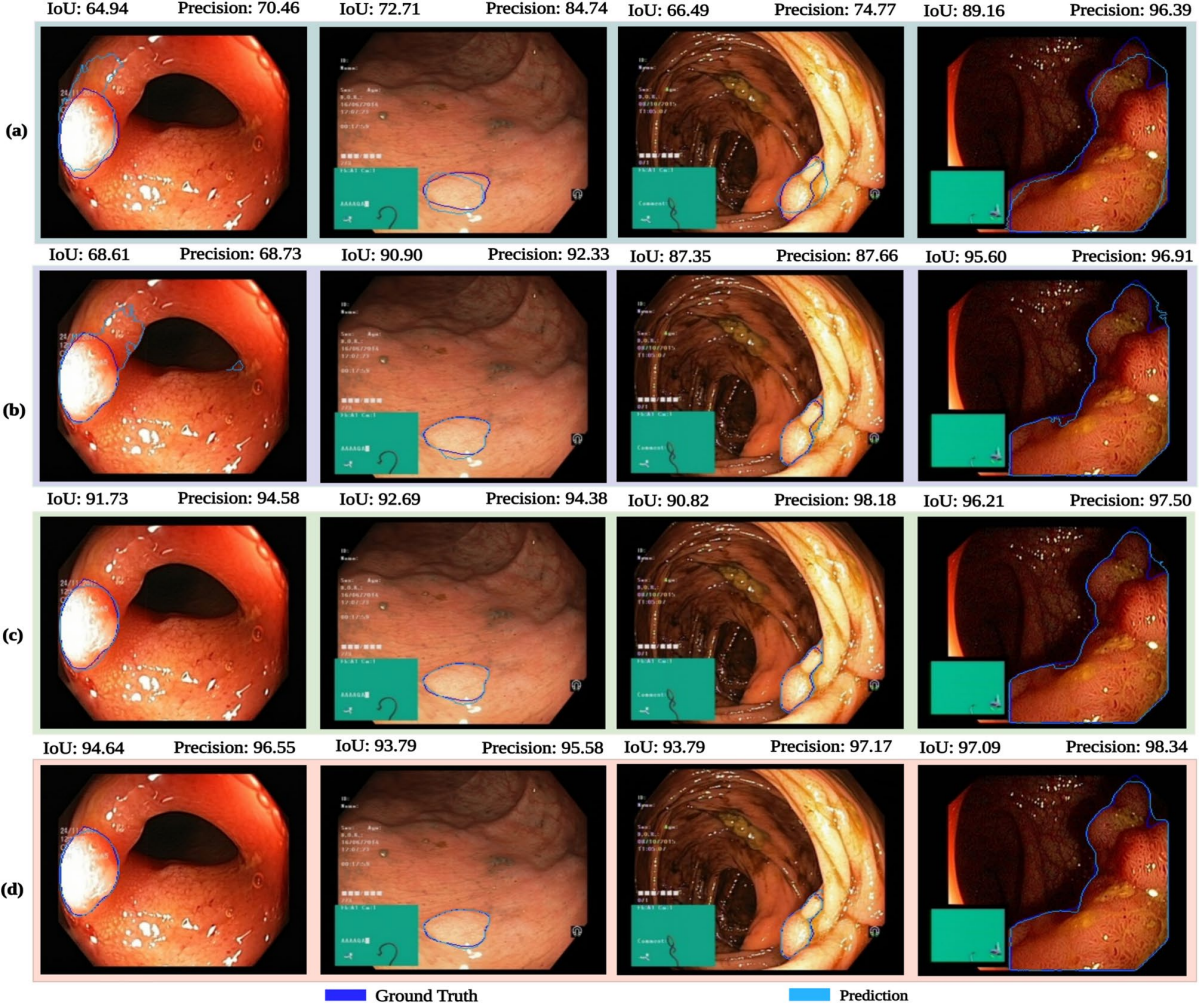
Visual comparison of segmentation results after progressive integration of architectural modules. (**a**) Baseline results using a simple attention module. (**b**) Improvement observed after incorporating boundary information into the attention module. (**c**) Further enhancement with the addition of the ASPP module for multiscale feature extraction. (**d**) Final results after integrating the boundary-aware loss function. The overall performance consistently improves with each module addition.

**Fig. 3:**
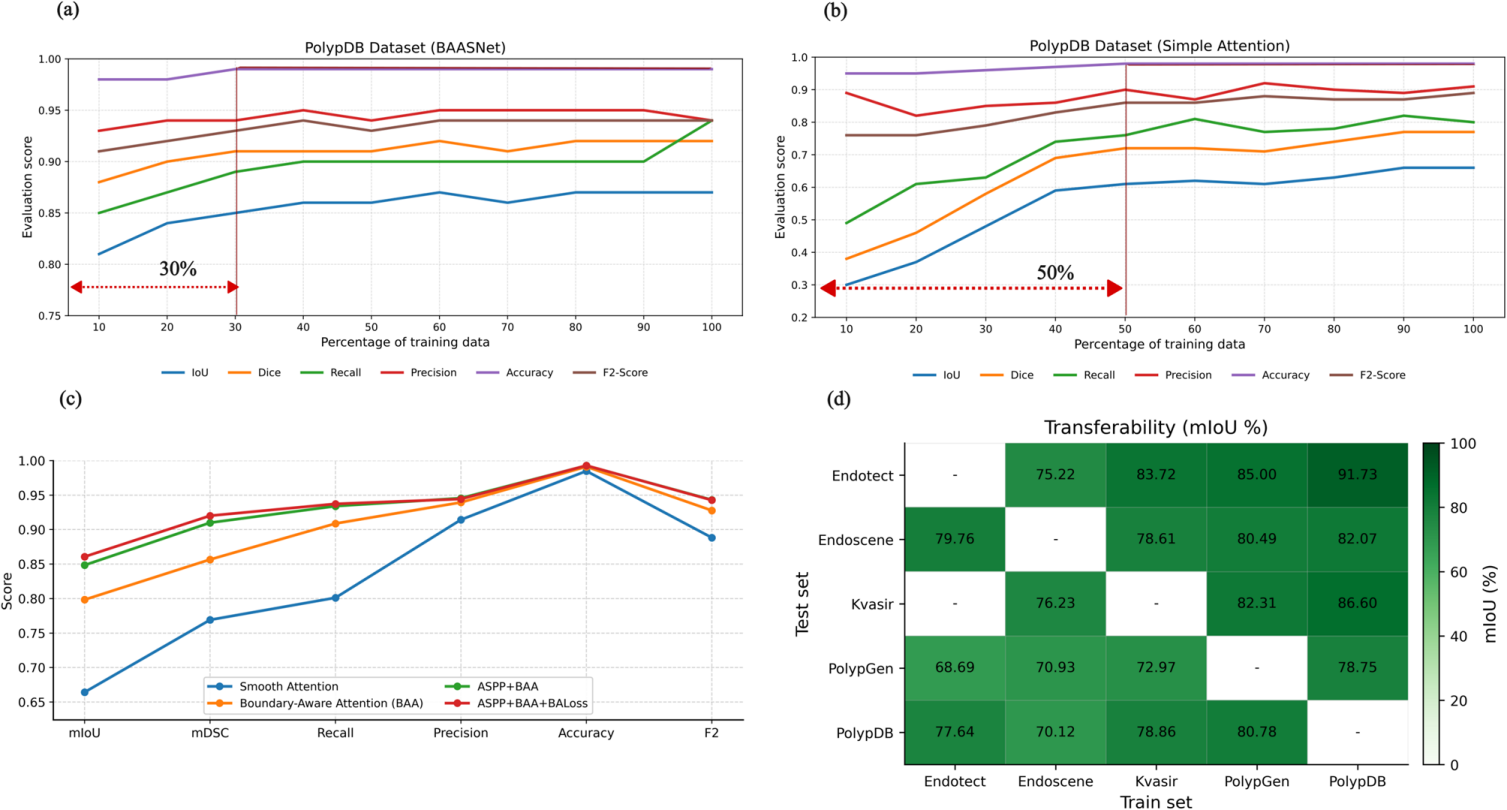
(**a**) Label-efficiency curve for BAASNet on the PolypDB dataset: the network requires approximately *30%* of the training data to reach its target performance (indicated by the dashed maroon marker). (**b**) Label-efficiency curve for the Smooth-Attention Net on PolypDB: the network requires approximately *50%* of the training data to reach its target performance, indicating lower data efficiency compared to BAASNet. (**c**) Ablation study illustrating the performance impact of progressively adding architectural components: Smooth Attention, Boundary-Aware Attention (BAA), Atrous Spatial Pyramid Pooling (ASPP) with Boundary-Aware Attention (BAA), and boundary-aware Loss. The results demonstrate consistent performance improvements with each addition. (**d**) Cross-dataset generalization analysis showing the performance (mIoU) of the proposed model when trained on one dataset and tested on others. The strong transferability highlights the model’s robustness across diverse domains and imaging modalities. To avoid data leakage, the model trained on the EndoTect dataset was not tested on the Kvasir test set.

To evaluate the impact of different components in the network, an ablation study was conducted across five imaging modalities, assessing performance after each architectural addition. The average results are presented in Figure 3 (c). First, the performance of the baseline model, which employs a simple Smooth Attention mechanism^29^, is shown. A clear improvement is observed when boundary information is incorporated into the attention module, underscoring the value of spatial boundary cues for refining segmentation. Polyp size variability is also found to negatively affect performance; therefore, an Atrous Spatial Pyramid Pooling (ASPP) module is integrated to better capture multi-scale contextual information, leading to a noticeable accuracy gain. Nevertheless, boundary regions remain challenging in some cases. To further improve boundary delineation and address class imbalance, a boundary-aware hybrid loss combining boundary-enhanced Dice, Binary Cross-Entropy (BCE), and edge-based BCE is introduced, yielding additional improvements. Overall, these findings help mitigate a common limitation in prior polyp segmentation work where boundaries are over-smoothed or confused with surrounding mucosa under challenging imaging conditions. Complete quantitative results are provided in (Supplementary Table 8). Qualitative results are presented in Figure 2. In the first row (a), the baseline model’s results are shown, where poor performance is evident around the boundaries, with the model confusing background regions with polyps. In the second row (b), clear improvements appear when boundary information is incorporated into the attention module. The third row (c) presents the results after adding the ASPP module, where further improvements can be seen, particularly in the first and second images. However, performance around polyp boundaries remained suboptimal. To overcome this, the boundary-aware loss function was introduced, as shown in the last row (d), leading to consistent performance even in complex scenarios where boundaries are indistinct and blend into the background.

**Table 4:** Generalizability evaluation of BAASNet. The model was trained exclusively on the predefined PolypDB training split and evaluated without further fine-tuning. Results are reported for unseen images from the predefined PolypDB test split, grouped by acquisition center, and for five external datasets that were not used during training. Within each evaluation setting, the best-performing result in each column is highlighted in bold.

Dataset/Test subset	mIoU	mDSC	Recall	Precision	Accuracy	F2
PolypDB test split grouped by acquisition center						
BKAI	*0.9061 ± 0.001*	*0.9467 ± 0.001*	*0.9635 ± 0.001*	*0.9685 ± 0.001*	*0.9960 ± 0.0002*	*0.9675 ± 0.001*
Karolinska	$\boldsymbol{0.9717 \pm 0.002}$	$\boldsymbol{0.9857 \pm 0.001}$	$\boldsymbol{0.9789 \pm 0.007}$	$\boldsymbol{0.9924 \pm 0.005}$	$\boldsymbol{0.9987 \pm 0.0001}$	$\boldsymbol{0.9897 \pm 0.003}$
Simula	*0.8592 ± 0.006*	*0.9133 ± 0.004*	*0.8860 ± 0.004*	*0.9514 ± 0.001*	*0.9841 ± 0.001*	*0.9375 ± 0.002*
External datasets not used during training						
ETIS	*0.7973 ± 0.007*	*0.8650 ± 0.008*	*0.9042 ± 0.027*	*0.9102 ± 0.008*	*0.9916 ± 0.001*	*0.9088 ± 0.006*
CVC-300	$\boldsymbol{0.8625 \pm 0.003}$	$\boldsymbol{0.9218 \pm 0.003}$	$\boldsymbol{0.9245 \pm 0.010}$	*0.9317 ± 0.007*	$\boldsymbol{0.9952 \pm 0.0002}$	$\boldsymbol{0.9302 \pm 0.004}$
CVC-ColonDB	*0.6769 ± 0.023*	*0.7530 ± 0.029*	*0.5531 ± 0.032*	*0.8968 ± 0.018*	*0.9619 ± 0.001*	*0.7970 ± 0.004*
CVC-ClinicDB	*0.7933 ± 0.010*	*0.8556 ± 0.009*	*0.7461 ± 0.011*	$\boldsymbol{0.9450 \pm 0.006}$	*0.9763 ± 0.001*	*0.8972 ± 0.006*
PolypGen	*0.7442 ± 0.120*	*0.8035 ± 0.119*	*0.8637 ± 0.045*	*0.8799 ± 0.084*	*0.9809 ± 0.005*	*0.8760 ± 0.078*

*Label efficiency for polyp segmentation:* To evaluate label efficiency, which refers to the amount of labeled training data required to achieve the target performance level for polyp segmentation, the model was trained on varying portions of the datasets. As shown in Figure 3 (a, b), BAASNet demonstrates superior label efficiency compared to the Smooth Attention network. On the PolypDB dataset, Unlike the simple model, which stabilizes around 50–*70%*, the proposed model achieves a performance plateau much earlier (approximately *30%*). These findings highlight the potential of BAASNet to perform effectively in scenarios with limited annotations, helping to address the challenge of data scarcity. The quantitative results are provided in (Supplementary Table 2 and Supplementary Table 3). Label efficiency was also evaluated on the combined dataset, where BAASNet required only *55%* of the available data to reach the target performance level. The complete quantitative results are presented in (Supplementary Table 4).

*Generalizability of the BAASNet for polyp segmentation:* To evaluate generalizability, BAASNet was trained only on the PolypDB training set and evaluated without fine-tuning on the test sets of each PolypDB center, including BKAI, Karolinska, and Simula. The model was also evaluated on the test sets of ETIS, CVC-300, CVC-ClinicDB, and CVC-ColonDB, and on the complete PolypGen dataset. PolypDB contains images from multiple centers and modalities, including BLI, FICE, LCI, and WLI^14^. As shown in Table 4, BAASNet maintains strong performance across datasets, indicating robust generalization across centers and imaging conditions. For completeness, representative challenging cases are shown in (Supplementary Figure 2).

To examine domain transfer capabilities, cross-dataset evaluations were performed. As shown in Figure 3 (d), the model trained on PolypDB was tested on four external datasets, and vice versa. In all cases, the model demonstrated consistent performance across test domains, further validating its cross-domain generalizability. Notably, to prevent data leakage, the Kvasir test set was excluded from evaluations involving models trained on the EndoTect dataset, since Kvasir images are included in its training set. Full quantitative results are provided in (Supplementary Table 5). The networks trained on each dataset were also evaluated on a unified test set consisting of the combined test splits from all datasets. The results are presented in (Supplementary Table 6). The strong performance indicates that the model maintains high accuracy even when images from different modalities and imaging conditions are combined at inference time. To further assess generalizability under diverse scenarios, we additionally trained and evaluated the network by progressively mixing the training and test sets. The corresponding results are reported in (Supplementary Table 9), demonstrating that the model preserves robust performance when data from diverse modalities and imaging conditions are jointly used.

To assess reproducibility, we repeated training and evaluation three times under identical experimental settings and dataset splits, varying only the random seed for initialization and mini-batch ordering. Detailed per-run results across all evaluated datasets are reported in Supplementary Table 17, while run-wise stability on the PolypGen dataset is visualized in Supplementary Figure 3. The results demonstrate stable performance with low sensitivity to random initialization. We also evaluated backbone choice by training and testing with ResNet-50, ResNet-101, and ConvNeXt-Base; ResNet-101 achieved the best overall overlap performance (mIoU/mDSC) (Supplementary Table 14). This stability, together with improved boundary delineation, suggests potential for robust real-time CADe margin visualization across modalities and centers.

In addition to segmentation accuracy and reproducibility, the computational cost of the proposed model was assessed. For an input resolution of *512 × 512*, BAASNet contains 96.64M trainable parameters and requires 309.53 GFLOPs per forward pass. On a single NVIDIA GeForce RTX 4090 GPU, the model achieves an average inference latency of 16.5–21.5 ms per image.

Extensive experiments confirm the effectiveness of the proposed network; however, certain challenging cases remain. As illustrated in the third image of the third column and the fourth image of the fourth column in Fig. 1, the method may produce under-segmentation in the presence of blood stains and when the polyp boundaries are poorly defined due to low contrast between the polyp and the surrounding mucosa. Future work will focus on improving performance in these cases through stronger global-context modeling.

## Methodology

**Fig. 4:**
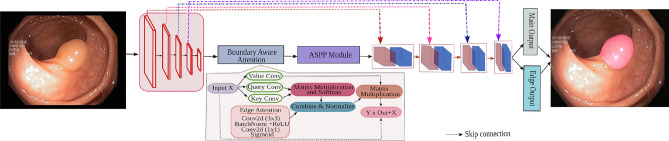
System overview of the proposed BAASNet architecture. The pipeline includes a backbone encoder, boundary-aware attention module, ASPP block, and a decoder producing segmentation and auxiliary edge maps.

Figure 4 illustrates the proposed pipeline for polyp segmentation. The architecture comprises four main components: a backbone, a boundary-aware attention module, an Atrous Spatial Pyramid Pooling (ASPP) module, and a decoder block.

### Backbone network

The backbone functions as the primary feature extractor, capturing both low-level and high-level representations from the input images. In the proposed model, a pretrained ResNet-101, a widely-used convolutional neural network, serves as the backbone. ResNet-101 is composed of five encoding blocks that progressively downsample the input, thereby increasing the receptive field and enhancing feature richness. The feature channels expand through the layers as follows: 64, 256, 512, 1024, and 2048. Residual skip connections help prevent vanishing gradients and support stable training. ResNet-101 is selected as the backbone because it learns subtle colonoscopy cues (textures, edges, small color changes) reliably with typical medical dataset sizes, while providing a good balance between performance and computational cost compared to lighter or transformer backbones.

### Boundary-aware attention(BAA)

This module improves segmentation accuracy by refining object boundaries through an edge-conditioned attention mechanism. From input feature maps, it constructs query, key, and value projections, computes an attention map via matrix multiplication and softmax, and integrates boundary cues using a dedicated Edge Attention branch. This branch applies convolution, batch normalization, and ReLU, followed by a sigmoid to generate an edge probability map. The final attention output is modified using the edge map, as shown in Equation 1:

1 $$\begin{aligned} \text {Output} = \text {Attention} \times (1 + \beta \times \text {EdgeProbabilityMap}) \end{aligned}$$

where *β* controls the strength of boundary information. The result is renormalized and fused with the input through a residual connection, as illustrated in Equation 2:

2 $$\begin{aligned} \text {Output} = \gamma \times \text {Output} + x \end{aligned}$$

Here, the parameter *γ* modulates the contribution of the attention-enhanced output. Both *β* and *γ* are implemented as learnable scalar factors, initialized to zero, and optimized using AdamW and standard backpropagation. Unlike prior boundary-aware modules^22^, which typically gate feature maps by directly multiplying them with boundary masks, our BAA introduces two key innovations. First, boundary cues modulate the attention map itself through a learnable scaling factor *β*, allowing dynamic adjustment of edge influence. Second, the residual connection preserves interior semantics while sharpening edges. Together, these choices transform boundary attention into a context-preserving, edge-conditioned self-attention mechanism.

### ASPP module

To capture multiscale contextual information, we employ an Atrous Spatial Pyramid Pooling (ASPP) module^30^. It processes the input through five parallel branches: a *1× 1* convolution, three *3× 3* atrous convolutions with dilation rates of 6, 12, and 18, using the same configuration as DeepLabv3+^30^, and a global average pooling branch to incorporate global context. The outputs from all branches are concatenated along the channel dimension and fused using a *1× 1* convolution. The detailed architecture is shown in (Supplementary Figure 1).

### Decoder

The decoder module progressively upsamples the encoded feature maps to reconstruct the segmentation map while incorporating skip connections from the encoder. Transposed convolutions are used for upsampling, followed by batch normalization and ReLU activation. A *3× 3* convolution is applied to refine the feature representations. An auxiliary edge detection branch, comprising convolutional layers, batch normalization, and ReLU activation, is incorporated to further enhance boundary localization. The network outputs both the final segmentation map and the boundary prediction map. The dual-task learning approach, segmentation, and edge detection enable the model to learn complementary features. It is important to note that during inference, only the segmentation maps are produced.

### Loss function

The loss function used in this study is defined in Equation 3. This loss function is inspired by^22^.

3 $$\begin{aligned} \begin{aligned} L=\alpha L_{BCE} + \beta L_{B-DICE} + \gamma L_{EdgeBCE} \end{aligned} \end{aligned}$$

where $L_{BCE}$ is the binary cross-entropy loss, $L_{B\text {-}DICE}$ is the boundary-enhanced Dice loss, and $L_{EdgeBCE}$ is the edge-based BCE loss. The weighting coefficients *α*, *β*, and *γ* are set to 1.0, 1.0, and 0.5, respectively, to balance dense supervision (BCE), region overlap (Dice), and boundary refinement (EdgeBCE), yielding accurate segmentation with sharper polyp edges. In the hybrid loss, each component contributes to performance: the boundary-enhanced Dice emphasizes boundary regions to mitigate class imbalance, BCE provides uniform pixel-wise supervision, and edge-based BCE refines predictions near object borders. Together, these terms guide optimization toward boundary-aware segmentation. In the boundary-enhanced Dice loss, the loss is calculated between the ground truth and the prediction, incorporating boundary weights, as shown in Equation 4.

4 $$\begin{aligned} \begin{aligned} L_{B-DICE} = 1 - \frac{2 \sum _i p_i g_i w_i + \epsilon }{\displaystyle \sum _i p_i w_i + \sum _i g_i w_i + \epsilon } \end{aligned} \end{aligned}$$

where $w_{i}$ is the boundary weight for pixel *i*, calculated using Equation 5, and *ε* is a smoothing factor for numerical stability.

5 $$\begin{aligned} w_i = 1.0 + 5.0 \,\cdot \, \exp \!\Bigl (-\frac{(b_i - b_{\max })^2}{0.1}\Bigr ) \end{aligned}$$

where $b_{i}$ is the boundary strength at pixel *i* computed using the Sobel filter applied to the ground-truth mask, and $b_{\text {max}}$ is its image-wise maximum. This creates a weight map that emphasizes the boundary regions by assigning a base weight of 1.0 to all pixels and an additional weight (up to 5.0) near the boundaries; the constant 0.1 controls the decay width of this emphasis. This loss encourages the model to focus on boundary accuracy, reducing boundary blurriness and improving overall segmentation quality.

In the edge-based binary cross entropy loss, the loss is calculated between the ground truth edge label and predicted edge probability obtained from the network, as shown in Equation 6, in addition to the main segmentation output.

6 $$\begin{aligned} \begin{aligned} L_{EdgeBCE} =-\frac{1}{n}\sum _{i=1}^{n}(G_{i} \cdot log(\hat{G}_{i})+(1-{G}_{i}) \cdot log(1-\hat{G}_{i})) \end{aligned} \end{aligned}$$

where n is the number of pixels, $G_{i}$ is the ground truth edge label for pixel i, $\hat{G}_{i}$ is the predicted edge probability for pixel i [0,1], $G_{i}$ is calculated using the Equation 7.

7 $$\begin{aligned} \begin{aligned} G_{i}=1\cdot (\sqrt{(S_{x}*g_{i})^{2}+(S_{y}*g_{i})^{2}}>0.1) \end{aligned} \end{aligned}$$

Here, $S_{x}$ and $S_{y}$ are the Sobel operators in the x and y directions, * denotes the convolution operation, and *1·* is the indicator function (1 if the condition is true, otherwise 0), and 0.1 is the threshold value.

### Experimental details

All models used in this study are implemented using the PyTorch framework. For each dataset, the default train-test splits provided by the respective sources are utilized. All images and their corresponding masks are resized to 512 *×* 512 pixels prior to input into the network. To enhance generalization, the training set is augmented using techniques such as flipping, rotation, Gaussian blur, and shift-scale transformations. All models are trained for 300 epochs. The AdamW optimizer is used with an initial learning rate of *1e-4* and a batch size of 16 (effective batch size 32 using gradient accumulation over 2 steps) during training. To comprehensively evaluate the proposed model, six performance metrics are employed: mean Intersection over Union (mIoU), mean Dice similarity coefficient (mDSC), Recall, Precision, F2-score, and Accuracy. The F2-score is used in this study due to its suitability for medical applications, as it assigns a higher weight to Recall than Precision, thereby reducing the risk of false negatives.

## Conclusion

In this paper, a novel end-to-end segmentation framework is presented, incorporating boundary-aware attention and an ASPP module. This combination effectively improves boundary precision and contextual representation, leading to high-quality segmentation masks. Extensive experiments on nine publicly available datasets demonstrate that the proposed method achieves strong and competitive performance in terms of mIoU and mDSC across diverse datasets and imaging modalities. The model also shows strong generalization across datasets and imaging modalities and demonstrates label efficiency by achieving high accuracy with reduced amounts of training data. Furthermore, the method exhibits robustness in challenging scenarios, including low contrast and complex polyp boundaries, highlighting its potential for future integration into real-time clinical decision-support systems. Despite these advancements, opportunities for further improvement remain. Future work will focus on enhancing global contextual modeling through the integration of Transformer-based modules to further improve segmentation accuracy in complex clinical scenarios.

## Supplementary Information

Supplementary Information.

## Data Availability

The source code for BAASNet is available on GitHub at https://github.com/Khola-naseem/BAASNet.git and has been archived on Zenodo at https://doi.org/10.5281/zenodo.21357538. All datasets used in this work are publicly accessible: Kvasir-SEG: https://datasets.simula.no/downloads/kvasir-seg.zip. PolypDB: https://osf.io/pr7ms/. CVC-EndoSceneStill: https://www.kaggle.com/datasets/kholanaseem/endoscene/data. PolypGen: https://doi.org/10.7303/syn26376615. ETIS, CVC-300, CVC-ClinicDB, CVC-ColonDB: https://www.kaggle.com/datasets/kholanaseem/testset. EndoTect: https://www.kaggle.com/datasets/debeshjha1/endotect-dataset.
