# Supplementary material for: BAASNet: boundary-aware deep learning for accurate polyp segmentation in colonoscopy
Source: Sci Rep. 2026 Aug 5;16:24170. doi: 10.1038/s41598-026-65397-5 (PMC13443481; doi:10.1038/s41598-026-65397-5)
Supplement: Supplementary file 1 [file 41598_2026_65397_MOESM1_ESM.pdf]

Supplementary information

BAASNet: Boundary-Aware Deep Learning for Accurate Polyp Segmentation in Colonoscopy

---

Supplementary Note: Detailed Module description

ASPP Module:

To capture multiscale contextual information, the ASPP module is integrated after the Boundary Attention Module. In the ASPP module, atrous (dilated) convolutions are used. The ASPP module helps expand the receptive field without increasing the number of parameters or losing spatial resolution, which is why it has become a standard component in many segmentation architectures. The detailed architecture is presented in Supplementary Figure 1. In ASPP, the major component is the atrous convolution, in which the dilation rate is used to control the spacing between kernel elements. Multiple parallel atrous convolutions enhance detection Accuracy by analyzing faults from various perspectives.

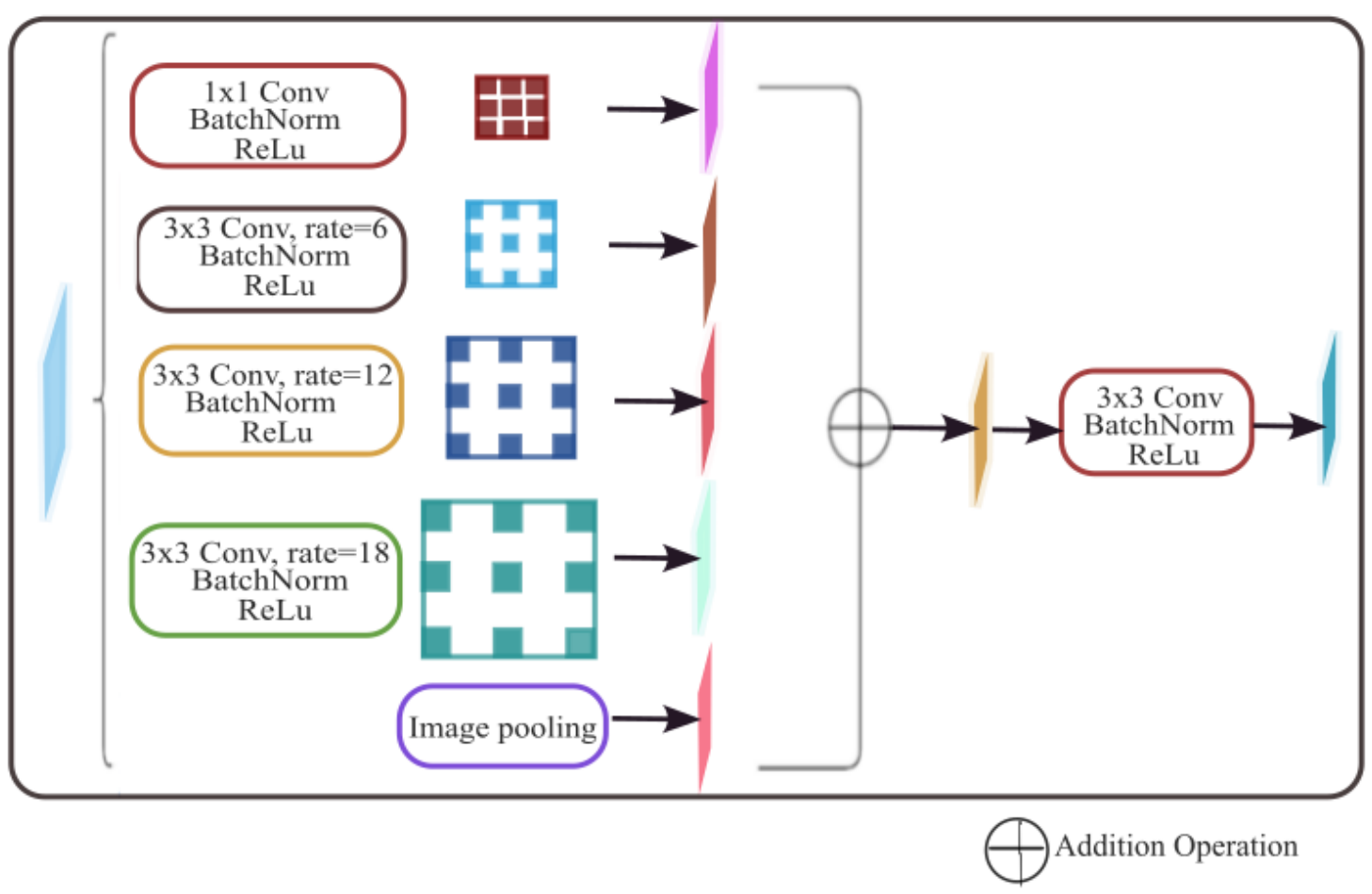

Supplementary Figure 1. The systematic overview of Atrous Spatial Pyramid Pooling (ASPP). The pipeline includes a parallel dilated convolutions with the rate 6,12,18, batch normalization, ReLu, image pooling and 1×1 convolutions.

We use dilation rates (6,12,18) to capture context at three increasingly larger receptive fields while preserving sufficient spatial detail. This configuration follows the ASPP design in DeepLabv3+, where it has been empirically shown to perform well for semantic segmentation at an output stride of 16. Multiscale feature extraction is particularly useful in our case, in which polyps of varying sizes and shapes are present. In polyp detection, this multiscale feature extraction allows the network to robustly localize and delineate polyps of diverse sizes and morphologies. Additionally, within ASPP, adaptive average pooling with an output size of 1 is used to capture global contextual information, which helps detect polyps effectively even in complex backgrounds.

Supplementary Note: Detailed supplementary experiment description

Data-efficiency experiments. Label efficiency was assessed by dividing the PolypDB training set into percentage-based subsets, while the test set remained unchanged (see details in Supplementary Table 1). As detailed in Supplementary Table 2, training the model with only 30% of the dataset yields performance comparable to training with 100%. Using only 10% of the training data, the model achieves 93.91% of the full-data IoU and 95.33% of the full-data Dice.

Supplementary Table 1. PolypDB training-set subsets used for the label-efficiency study: number of images per percentage split (test set unchanged).

Percentage	No of images
10	354
20	708
30	1062
40	1415
50	1769
60	2123
70	2476
80	2830
90	3184

Supplementary Table 2. BAASNet performance on PolypDB when trained with different fractions of the training data; metrics reported on the fixed test set (showing near saturation by ~50%).

Metrics	10	20	30	40	50	60	70	80	90	100
**IoU**	0.8146	0.8385	0.8542	0.8570	0.8612	0.8651	0.8632	0.8670	0.8671	0.8674
**Dice**	0.8772	0.8967	0.9089	0.9096	0.9139	0.9157	0.9149	0.9193	0.9185	0.9201
**Recall**	0.8476	0.8698	0.8927	0.8997	0.8999	0.9017	0.9005	0.9008	0.9006	0.9373
**Precision**	0.9261	0.9374	0.9437	0.9486	0.9410	0.9472	0.9472	0.9455	0.9501	0.9443
**Accuracy**	0.9808	0.9835	0.9859	0.9870	0.9863	0.9870	0.9869	0.9868	0.9872	0.9930
**F2 Score**	0.9093	0.9230	0.933	0.9384	0.9325	0.9378	0.9375	0.9362	0.9398	0.9428

Under the same evaluation protocol, a simple attention network reaches near table performance with 50% of the data (Supplementary Table 3). Using only 10% of the training data, the model achieves 45% of the full-data IoU.

Supplementary Table 3. Simple Attention baseline trained on varying fractions of PolypDB; comparison to its own 100%-data performance

	10	20	30	40	50	60	70	80	90	100
**IoU**	0.2996	0.3695	0.4775	0.5872	0.60760	0.6214	0.61470	0.6304	0.6633	0.6643
**Dice**	0.3786	0.4564	0.5828	0.6899	0.71590	0.7219	0.71320	0.7383	0.7687	0.7691
**Recall**	0.4867	0.6060	0.6275	0.7423	0.76220	0.8112	0.77400	0.7845	0.8172	0.8012
**Precision**	0.8918	0.8171	0.8467	0.8594	0.89680	0.8731	0.91890	0.9017	0.8865	0.9144
**Accuracy**	0.9499	0.9537	0.9575	0.9668	0.98250	0.9833	0.98270	0.9838	0.9848	0.9849
**F2 Score**	0.7645	0.7639	0.7914	0.8332	0.86210	0.8574	0.88230	0.8719	0.8712	0.8884

Mixed-dataset sampling. We additionally partitioned a combined dataset (Kvasir, PolypDB, Endoscene, Endotect) in 10% increments and report percentages relative to training on 100% of the data (i.e., the fully supervised baseline = 100%). With only 10% of the data, our model reaches 95.55% of the baseline IoU and 96.96% of the baseline Dice; Precision is 98.17%, recall 93.43%, Accuracy 99.2%, and F2 97.1% of their full-data values. Overall, these results indicate strong data efficiency across heterogeneous sources and image characteristics, with near-saturation of full-data performance well before using the entire dataset (Supplementary Table 4).

Supplementary Table 4. BAASNet trained on mixed data (Kvasir, PolypDB, EndoScene, PolypGen) at increasing fractions; test protocol fixed to assess data efficiency across heterogeneous sources.

	10	20	30	40	50	60	70	80	90	100
**IoU**	0.8227	0.8400	0.8462	0.8509	0.8545	0.8542	0.8596	0.8578	0.8583	0.8610
**Dice**	0.8830	0.8960	0.9000	0.9033	0.9064	0.9066	0.9097	0.9093	0.9094	0.9106
**Recall**	0.8296	0.8654	0.8808	0.8869	0.8882	0.8707	0.8800	0.8928	0.8933	0.8879
**Precision**	0.9349	0.9331	0.9355	0.9385	0.9430	0.9540	0.9512	0.9439	0.9455	0.9523
**Accuracy**	0.9746	0.9781	0.9800	0.9810	0.9816	0.9809	0.9816	0.9822	0.9824	0.9826
**F2-Score**	0.9117	0.9187	0.9240	0.9277	0.9315	0.9361	0.9361	0.9332	0.9346	0.9387

Cross-dataset generalization. To assess generalization, the model was trained on one dataset and evaluated on the test sets of the others (Supplementary Table 5), including: trained on PolypDB → tested on other datasets; trained on Endotect → tested on other datasets; trained on Kvasir → tested on other datasets; trained on PolypGen → tested on other datasets: trained on Endoscene → tested on other datasets. Across all transfers, Accuracy remains ≥94.59% (max 98.52%), indicating robust generalization despite domain shifts.

Supplementary Table 5. Cross-dataset generalization: train on one dataset (PolypDB, EndoTect, Kvasir, EndoScene) and evaluate on the others; mean Dice, Recall, Precision, F2, and Accuracy reported.

Network train on PolypDB Tested on other Datasets					
	**mDSC**	**Recall**	**Precision**	**F2**	**Accuracy**
**Endoscene**	88.03	74.47	96.66	91.22	97.40
**Endotect**	95.01	93.39	96.29	95.70	98.52
**Kvasir**	91.53	87.41	96.41	94.47	97.52
**PolypGen_Complete**	81.23	86.90	89.23	88.76	98.08
**PolypGen_C6**	84.38	85.76	94.13	92.33	98.44
**Network train on EndoTect Tested on other Datasets**					
	**mDSC**	**recall**	**Precision**	**F2**	**Accuracy**
**PolypDB**	83.91	85.96	88.26	87.79	97.77
**PolypGen _C6**	74.19	91.77	77.21	79.74	97.19
**EndoScene**	86.68	78.94	91.79	88.89	97.4
**Network train on Kvasir Tested on other Datasets**					
	**mDSC**	**recall**	**Precision**	**F2**	**Accuracy**
**PolypDB**	85.11	86.16	90.35	89.48	97.98
**EndoScene**	85.4	77	93.52	89.67	97.38
**PolypGen_C6**	78.86	83.49	87.14	86.38	97.71
**Network train on EndoScene Tested on other Datasets**					
	**mDSC**	**recall**	**Precision**	**F2**	**Accuracy**
**PolypDB**	77.78	73.78	85.72	83.03	96.63
**EndoTect**	83.32	75.75	87.31	84.72	94.89
**Kvasir**	83.93	78.6	85.61	84.11	94.59
**PolypGen_C6**	76.72	79.51	92.71	89.73	97.88
**Network train on PolypGen Tested on other Datasets**					
	**mDSC**	**recall**	**Precision**	**F2**	**Accuracy**
**PolypDB**	86.92	86.04	92.99	91.51	98.21
**EndoTect**	90.8	86.76	92.69	91.44	97.09
**Kvasir**	88.29	83.42	93.09	90.98	96.44
**Endoscene**	87.11	73.77	95.61	90.26	97.26

Combined test-set evaluation. We further trained on a single dataset and evaluated on the combined test set of all remaining datasets (Supplementary Table 6). Across all settings, performance exceeds 96%, indicating robust adaptability to diverse imagery.

Supplementary Table 6. Combined test-set evaluation: single-dataset training (PolypDB, EndoScene, Kvasir) with testing on the union of all remaining datasets.

Trained network	mIoU	mDSC	Recall	Precision	F2	Accuracy
**PolypDB_Trained_Network**	86.19	91.13	87.47	96.09	94.23	98.21
**EndoScene_Trained_Network**	74.14	81.52	76.19	87.77	85.18	96.17
**Kvasir_Trained_Network**	80.42	86.51	85.68	91.49	90.26	97.51
**PolypGen_Trained_Network**	81.50	87.63	83.74	93.17	91.12	97.51

A model trained on PolypDB unseen images of the different datasets, strong results demonstrating generalization to broader imaging conditions (Supplementary Table 7). Qualitative results are presented in Supplementary Figure 2.

Supplementary Table 7. PolypDB-trained network evaluated on ETIS, CVC-300 , CVC-ColonDB, CVC-Clinic, BKAI, Karolinska, PolypGen and Simula .

Datasets	mIoU	mDSC	Recall	Precision	Accuracy	F2
BKAI	89.52	93.97	96.97	97.14	99.70	97.11
Karolinska	96.38	98.14	98.59	98.69	99.74	98.67
Simula	94.65	97.08	97.68	98.23	99.60	98.12
ETIS	79.68	86.59	91.24	91.58	99.22	91.51
CVC-300	86.12	92.03	91.41	93.92	99.51	93.41
CVC-ColonDB	65.79	72.95	55.07	90.23	96.21	80.01
CVC-Clinic	78.62	84.92	73.34	94.23	97.51	89.15
PolypGen	73.93	79.77	85.64	87.95	98.08	87.71

Ablation study. An incremental ablation (Supplementary Table 8) demonstrates that each architectural component contributes to performance gains, particularly for boundary handling and lesions of varying sizes. Starting from the Smooth Attention baseline, introducing Boundary Attention (BA) yields the largest single-step improvement, indicating that explicitly modeling edges is crucial for cleaner masks and fewer boundary-related errors. Adding ASPP on top of BAA further boosts recognition of lesions at different scales: recall and Precision rise together, and overall correctness increases. Finally, coupling ASPP+BAA with the boundary-aware loss reconciles the remaining Precision–recall trade-offs and lifts every summary metric.

Supplementary Table 8. Ablation study: incremental contributions of Boundary aware Attention (BAA), ASPP, and boundary-aware loss on overall segmentation quality and boundary handling.

Network	mIoU	mDSC	Recall	Precision	Accuracy	F2
Smooth Attention	0.66428	0.76912	0.8012	0.91436	0.98496	0.88836
Boundary Aware Attention(BAA)	0.79840	0.85666	0.90884	0.93946	0.99122	0.92774

ASPP+BA	0.84850	0.91004	0.93406	0.94562	0.99286	0.94316
ASPP+BA+BALoss	0.86074	0.92014	0.937328	0.94434	0.99298	0.94276

Progressive train/test mixing. Finally, progressively combining training and test sources (Supplementary Tables 9) shows that the network sustains strong performance across varied imaging conditions and modalities.

Supplementary Table 9. Robustness under progressive train and test-set mixing: results on combined test sets (e.g., EndoScene+Kvasir, EndoScene+Kvasir+PolypDB, , EndoScene+Kvasir+PolypDB+PolypGen)

Dataset	mIoU	mDSC	Recall	Precision	F2	Accuracy
**Endoscene_kvasir_testset**	83.74	89.46	86.01	92.19	90.9	97.18
**Endoscene_kvasir_PolypDB_test**	86.69	91.69	88.59	95.11	93.7	98.18
**Endoscene_kvasir_PolypDB_PolypGen _test**	86.10	91.06	88.79	95.23	93.8	98.26

Complete Results. Complete results for our network on PolypDB are provided in Supplementary Table 10. Supplementary Table 11 presents the full results on Kvasir-SEG, EndoScene, and EndoTect.

Supplementary Table 10. PolypDB results by imaging modality (BLI, FICE, LCI, NBI, WLI): method-wise comparison including the proposed model.

Dataset	Methods	mIoU	mDSC	Recall	Precision	F2
**PolypDB(BLI)**	CaraNet	0.585	0.724	0.690	0.805	0.698
	TGANet	0.222	0.692	0.811	0.644	0.708
	PVTCASCADE	0.680	0.813	0.908	0.725	0.801
	DuAT	0.674	0.805	0.908	0.765	0.820
	SSFomerL	0.675	0.785	0.843	0.771	0.809
	Proposed Model	0.8179 ± 0.0099	0.8960 ± 0.0067	0.9351 ± 0.0026	0.8984 ± 0.0024	*0.9055 ± 0.0015*
**PolypDB(FICE)**	CaraNet	0.569	0.630	0.608	0.814	0.615
	TGANet	0.592	0.690	0.708	0.728	0.696
	PVTCASCADE	0.721	0.780	0.811	0.759	0.797
	DuAT	0.559	0.675	0.908	0.583	0.773
	SSFomerL	0.761	0.830	0.871	0.801	0.853
	Proposed Model	0.8389 ± 0.0050	0.9054 ± 0.0033	0.9608 ± 0.0056	0.9385 ± 0.0140	0.9428 ± 0.0107
**PolypDB(LCI)**	CaraNet	0.760	0.858	0.834	0.919	0.840
	TGANet	0.836	0.906	0.882	0.947	0.890
	PVTCASCADE	0.834	0.907	0.942	0.921	0.906
	DuAT	0.855	0.919	0.920	0.925	0.919

	SSFomerL	0.857	0.921	0.906	0.947	0.911
	Proposed Model	0.9075 ± 0.0041	0.9494 ± 0.0022	0.9598 ± 0.0142	0.9702 ± 0.0142	0.9680 ± 0.0085
**PolypDB(NBI)**	CaraNet	0.725	0.809	0.831	0.878	0.819
	TGANet	0.732	0.840	0.864	0.865	0.835
	PVTCASCADE	0.777	0.859	0.939	0.832	0.894
	DuAT	0.749	0.826	0.867	0.874	0.848
	SSFomerL	0.785	0.843	0.909	0.846	0.866
	Proposed Model	0.8594 ± 0.0170	0.9205 ± 0.0127	0.9382 ± 0.0224	0.9475 ± 0.0126	0.9455 ± 0.0056
**PolypDB(WLI)**	CaraNet	0.858	0.913	0.915	0.922	0.911
	TGANet	0.854	0.909	0.917	0.928	0.910
	PVTCASCADE	0.870	0.920	0.917	0.944	0.917
	DuAT	0.882	0.929	0.951	0.944	0.924
	SSFomerL	0.889	0.933	0.951	0.944	0.930
	Proposed Model	0.8741 ± 0.0062	0.9233 ± 0.0043	0.8983 ± 0.0048	0.9553 ± 0.0005	0.9434 ± 0.0015

Results by Polyp Size:

To analyze segmentation performance across different lesion scales, results are reported in Supplementary Table 12 for small, medium, and large polyps using the following area-based definition: small polyps have area > 5, medium polyps have area ≥ 5 and < 15, and large polyps have area > 15. The small-polyp size category and its reported values are taken from Caranet, which uses the same size-based characterization for small polyps, ensuring consistency with prior work. Overall, the table summarizes IoU and Dice scores per dataset and size group, highlighting how performance varies with polyp scale and how some datasets do not provide results for all size categories (shown as "–").

Supplementary Table 12. Segmentation performance across five datasets, reported as IoU and Dice scores stratified by polyp size (small, medium, large). Dashes (–) indicate size categories not available for that dataset.

**Dataset**	**Iou-small**	**Dice_small**	**Iou medium**	**Dice_medium**	**Iou_large**	**Dice_large**
**PolypDB BLI**	0.7919	0.837	-	-	-	-
**PolypDB FICE**	0.73	0.778	0.9596	0.979	-	-
**PolypDB LCI**	0.892	0.941	0.92	0.959	0.977	0.988
**PolypDB NBI**	0.785	0.827	0.901	0.951	0.945	0.972
**PolypDB WLI**	0.7851	0.831	0.91	0.94	0.87	0.91
**EndoTect**	0.75	0.78	0.89	0.93	0.84	0.90
**EndoScene**	0.85	0.89	0.90	0.94	0.63	0.73

**Kvasir-SEG**	0.476		0.9038	0.944	0.86	0.91
**PolypGen**	0.64	0.68	0.88	0.92	0.89	0.94

Supplementary Table 11. Method comparisons on Kvasir-SEG, EndoScene, and EndoTect: mIoU, mDSC, Recall, Precision, F2, and Accuracy for prior methods versus the proposed model.

**Dataset**	**Methods**	**mIoU**	**mDSC**	**Recall**	**Precision**	**F2**	**Accuracy**
**Kvasir-SEG**	DDANet	0.7800	0.8576	0.888	0.864	-	-
	NanoNet-A	0.728	0.823	0.859	0.837	0.84	0.946
	Polypseg+	0.707	0.7810	0.854	-	-	0.9408
	Duplex contextual relation	0.8444	0.9014	-	-	-	-
	ConvNeXt-Base+MPE	0.816	0.882	-	-	-	-
	Proposed Model	0.856± 0.007	0.908± 0.005	0.91± 0.008	0.925± 0.004	0.922± 0.005	0.9743± 0.002
**Endoscene**	Polypseg+	0.635	0.7170	0.765	-	-	0.949
	Duplex contextual relation	0.7886	0.8541	-	-	-	-
	Proposed Model	0.810± 0.008	0.874± 0.006	0.768± 0.009	0.94± 0.008	0.90± 0.007	0.974± 0.001
**Endotect**	NanoNet-A	0.650	0.751	0.824	0.774	0.78	0.926
	ConvNeXt-Base+MPE	0.797	0.857	-	-	-	-
	DDANet	0.7010	0.787	0.7987	0.8577	-	-
	Proposed Model	0.837 ± 0.004	0.8967 ± 0.003	0.8428 ± 0.017	0.9284± 0.019	0.9107 ± 0.010	0.9677 ± 0.001

Generalizability Failure analysis: When the model trained on PolypDB is tested on external datasets, the main errors are linked to unclear borders and appearance changes caused by strong glare, bubbles/foam, folds, and small or low-contrast polyps. The qualitative results are presented in Supplementary Figure 3. The first case contains visible blood on and

around the polyp, together with strong glare on the wet surface. These factors obscure the true boundary and reduce contrast, leading to mask leakage outside the polyp (false positive, red) and missed parts of the boundary (false negative, blue). In the second image, the polyp surface is smooth and affected by glare, and the boundary between the polyp and the surrounding tissue is subtle; this results in over-segmentation, where the predicted mask extends into nearby regions (red). The third image shows a polyp located on a folded, complex surface with similar surrounding texture and partial shadowing, causing under-segmentation, particularly in the upper region (blue). In the fourth image, the large polyp has an irregular shape with strong glare/overexposure and nearby bubbles/foam, making the outer boundary difficult to delineate and resulting in substantial under-segmentation (blue) with small spurious detections (red). The final example contains a small polyp with slight blur and low contrast, so the model captures only the most prominent part and misses a portion of the lesion (blue), producing an incomplete segmentation.

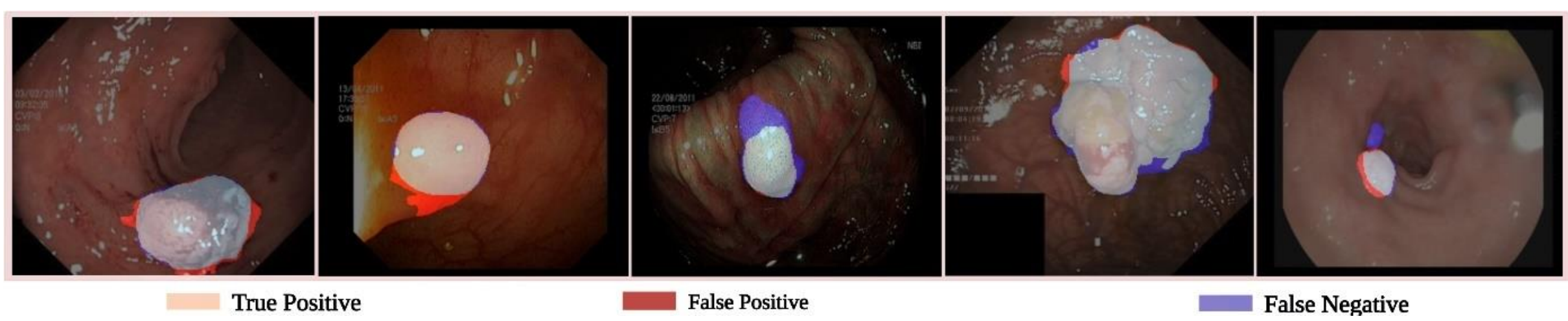

Supplementary Figure 2. Qualitative generalizability failure analysis of the proposed network. The network is trained on the PolypDB dataset and evaluated on external datasets. White regions indicate correctly segmented polyps, red regions denote false positives, and blue regions indicate false negatives.

PolypGen Complete generalization results:

Supplementary Table 13. Center-wise generalization performance of the proposed method on the PolypGen dataset.

Center	mIoU	mDSC	Recall	Precision	Accuracy	F2
C1	82.11	88.14	90.99	92.26	98.25	92.01
C2	74.13	79.12	83.46	84.66	97.33	84.41
C3	86.77	92.05	91.28	95.61	98.85	94.71
C4	56.01	60.85	78.32	75.44	97.43	75.99
C5	65.06	73.20	80.52	85.56	97.97	84.50
C6	78.75	84.38	85.76	94.13	98.44	92.33

Impact of Backbone Choice on BAASNet Performance

Supplementary Table 14 compares BAASNet performance on PolypDB using three backbone variants (ResNet-50, ResNet-101, and ConvNeXt-Base) under the same training and testing setting. Switching

from ResNet-50 to the deeper ResNet-101 improves most overlap-based metrics, with mIoU increasing from 85.61 to 86.40 and mDSC rising from 90.00 to 92.01, alongside a strong recall gain from 89.26 to 93.73 and an F2-score increase from 93.49 to 94.28. These gains suggest that the additional depth in ResNet-101 strengthens multi-level feature representation and boundary delineation, leading to better region agreement.

Interestingly, ConvNeXt-Base shows a different trade-off: it achieves the highest recall (94.59) and the highest accuracy (99.36), while maintaining a competitive F2-score (94.23), but its mIoU (85.64) and mDSC (90.68) remain below ResNet-101. This indicates ConvNeXt-Base is especially strong in sensitivity (detecting more true polyp pixels), whereas ResNet-101 provides the best overall overlap quality. Precision differences are minor across backbones (ResNet-50: 94.62, ResNet-101: 94.43, ConvNeXt-Base: 94.15) and are outweighed by the recall and overlap improvements, making ResNet-101 the best balanced choice for segmentation quality, while ConvNeXt-Base may be preferred when maximizing sensitivity is the priority.

Supplementary Table 14. Supplementary Table 14. BAASNet performance on PolypDB with different backbones (ResNet-50, ResNet-101, ConvNeXt-Base) under the same training/testing setting.

Method	mIoU	mDSC	Recall	Precision	Accuracy	F2
BAASNet -Resnet50	85.61	90.00	89.26	94.62	98.62	93.49
BAASNet-Resnet101	86.40	92.01	93.73	94.43	99.30	94.28
BAASNet-ConvNext_base	85.64	90.68	94.59	94.15	99.36	94.23

Dataset statistics:

The dataset statistics are presented in Supplementary Table 15. Since there is no universally accepted definition of small, medium, and large polyps, we categorize polyp size based on the percentage of image area. Specifically, small polyps occupy less than 5% of the image area, medium polyps range from 5% to 15%, and large polyps are those whose size is greater than 15% of the image area.

Supplementary Table 15. Dataset statistics including image counts, pixel-level class distribution, and class imbalance.

Dataset	num_images	num_images_with_polyp	polyp_pixels	background_pixels	pct_polyp_pixels	pct_background_pixels	imbalance_ratio
CVC-300	60	60	578743	16641257	3.360877	96.63912	28.75414
CVC-ClinicDB	62	62	547480	6309224	7.984594	92.01541	11.52412
CVC-ColonDB	380	380	8123114	100936886	7.448298	92.5517	12.42589
ETIS-Larib	216	216	10633801	223780879	4.53632	95.46368	21.0443

EndoScene	912	912	10691020	143091284	6.952048	93.04795	13.38425
EndoTect	200	200	14261707	79986101	15.13214	84.86786	5.608452
Kvasir-SEG	1000	1000	55191016	296859878	15.677	84.323	5.378772
PolypGen	1537	1411	199499279	2318741364	7.922169	92.07783	11.62281
PolypDB	3929	3929	222406765	2584992371	7.922164	92.07784	11.62281

Supplementary Table 16. Polyp size statistics and size-category distribution across datasets, reported as percentages of image area.

Dataset	median_size_pct	mean_size_pct	std_size_pct	min_size_pct	max_size_pct	pct_small_polyps	pct_medium_polyps	pct_large_polyps
CVC-300	2.773345	3.360877	2.553375	0.54669	18.41986	88.3333	10	1.666667
CVC-ClinicDB	4.545989	7.280071	8.015042	0.334563	45.87764	52.9411	33.82353	13.23529
CVC-ColonDB	3.61777	7.448298	10.06937	0.298258	63.15052	59.7368	28.68421	11.57895
ETIS-Larib	1.99962	4.321921	5.793978	0.10597	29.05421	75.6521	15.65217	8.695652
EndoScene	5.30122	7.633621	7.023481	0.168186	48.98275	47.463	38.90063	13.63636
EndoTect	10.85069	13.95748	10.51321	0.606283	59.05396	21.6346	41.82692	36.53846
Kvasir-SEG	10.40337	14.51318	13.01534	0.16837	81.23484	24.1768	40.45155	35.37159
PolypGen	4.082454	7.718997	9.70626	0.00162	74.03848	54.9876	30.23399	14.77833
PolypDB	2.727036	5.864276	8.615248	0.002332	75.24105	64.9897	23.36882	11.64148

Multiple-Run Consistency:

Supplementary Table 17 evaluates BAASNet on the all dataset across three independent runs using mIoU, mDSC, Accuracy, Precision, Recall, and F2-score. The results show highly consistent performance with only minor run-to-run variation, indicating that BAASNet is stable and reproducible. Supplementary Figure~3 visualizes the PolypGen results using mean $\pm$ standard deviation bars, highlighting the small run-to-run variability and reinforcing the overall robustness of the proposed method across repeated experiments.

Supplementary Table 17. BAASNet performance metrics on the all datasets across three experimental runs. The table reports IoU, Dice, Accuracy, Precision, Recall, and F2-score for Runs 1–3, showing consistently strong performance with only minor variability between runs.

Run	mIoU	mDSC	Recall	Precision	Accuracy	F2

PolypGen	0.7873	0.8471	0.8518	0.9473	0.9845	0.9265
PolypGen	0.7564	0.8143	0.9056	0.9233	0.9865	0.9197
PolypGen	0.7838	0.8436	0.879	0.9267	0.9849	0.9167
Kvasir-SEG	0.8493	0.9035	0.9039	0.9204	0.9728	0.9170
Kvasir-SEG	0.8633	0.9142	0.9203	0.9292	0.9766	0.9274
Kvasir-SEG	0.8560	0.9080	0.9050	0.9240	0.9736	0.9210
EndoScene	0.8162	0.8782	0.7668	0.9488	0.9746	0.9058
EndoScene	0.8011	0.8674	0.7589	0.9329	0.9727	0.8920
EndoScene	0.8130	0.8770	0.7772	0.9384	0.9747	0.9010
EndoTect	0.8370	0.8970	0.8616	0.9064	0.9670	0.9000
EndoTect	0.8336	0.8940	0.8280	0.9378	0.9671	0.9135
EndoTect	0.8407	0.8992	0.8389	0.9410	0.9690	0.9186
PolypDB	0.8623	0.9210	0.9414	0.9406	0.9930	0.9406
PolypDB	0.8563	0.9166	0.9327	0.9446	0.9928	0.9419
PolypDB	0.8601	0.9192	0.9413	0.9406	0.9931	0.9405

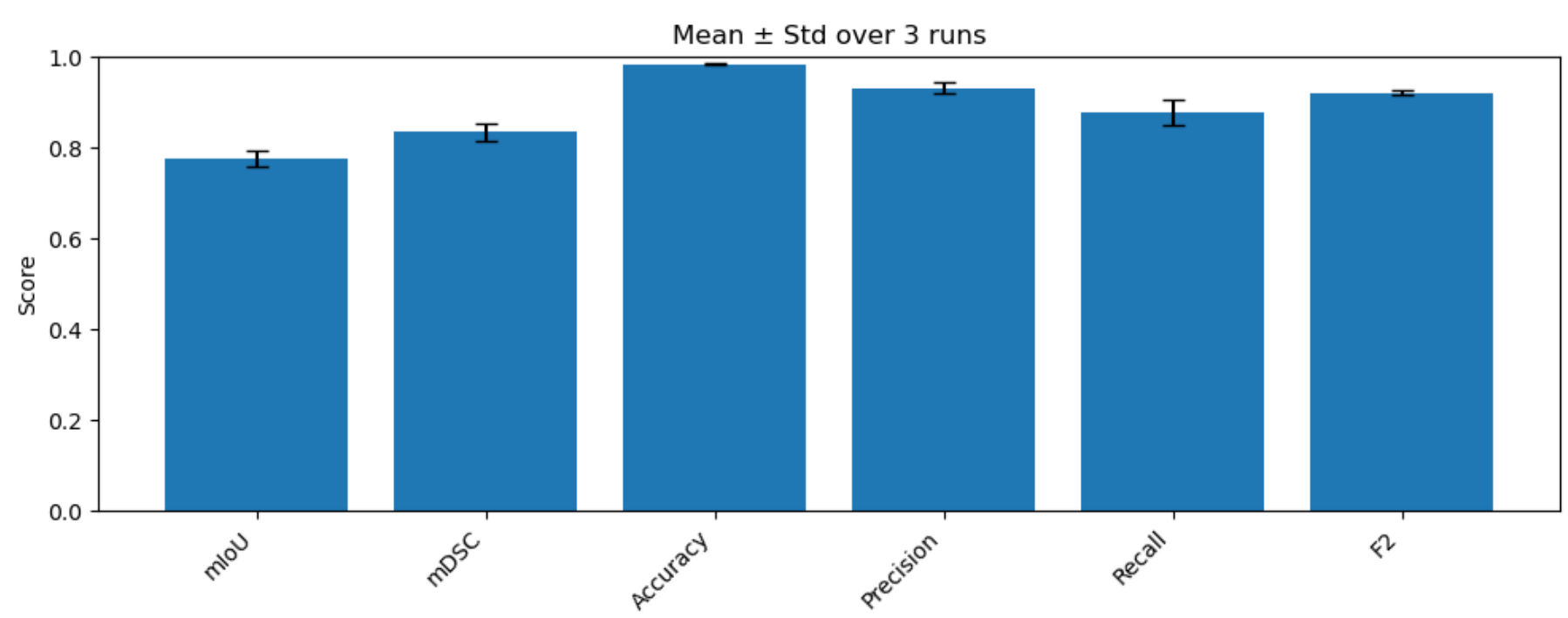

Supplementary Figure 3. Mean ± standard deviation of BAASNet performance on the PolypGen dataset over three runs. Bars show the average IoU, Dice, Accuracy, Precision, Recall, and F2-score, with error bars indicating variability across runs.

Statistical significance Testing:

Supplementary Table 18 presents a per-image statistical comparison between the baseline and the proposed method across IoU, Dice, Precision, Recall, and F2-score. The proposed method achieves substantial mean improvements over the baseline for all metrics, including +0.1804 IoU, +0.1344 Dice, +0.0856 Precision, +0.1664 Recall, and +0.1095 F2. These gains are further supported by non-overlapping, strictly positive 95% bootstrap confidence intervals for the mean differences (e.g., IoU: 0.1630–0.1988; Dice: 0.1172–0.1530), indicating that the improvements are robust at the dataset level. Statistical

hypothesis testing using both paired Wilcoxon signed-rank and paired t-tests yields extremely small p-values across all metrics (e.g., IoU: 1.17×10^{-61} / 4.17×10^{-62}), confirming that the performance gains are highly significant and unlikely to be due to random variation. Overall, these results demonstrate that the proposed method consistently and significantly outperforms the baseline across key segmentation quality measures.

Supplementary Table 18. Mean per-image segmentation metrics for baseline vs. proposed method, with mean improvement (New−Base), 95% bootstrap CI, and paired Wilcoxon and t-test p-values.

Metric	Mean (Base)	Mean (New)	Mean Diff (New-Base)	Boot 95% CI Low	Boot 95% CI High	Wilcoxon p	Paired t-test p
IoU	0.688832	0.869261	0.180429	0.162968	0.19881	1.17E-61	4.17E-62
Dice	0.786196	0.920645	0.134449	0.117245	0.152978	5.64E-40	1.87E-61
Precision	0.855166	0.940794	0.085628	0.066754	0.106363	2.35E-16	2.53E-31
Recall	0.755064	0.921487	0.166423	0.146947	0.186831	6.36E-47	2.42E-60
F2	0.818208	0.927695	0.109488	0.092113	0.128366	4.85E-28	3.77E-53

Real-Time Integration and Clinical Deployment Considerations:

BAASNet achieves real-time inference (512×512, single GPU) and performs well across nine multi-modal datasets. However, further validation on video-based benchmarks such as ENDOTEST [1], designed for evaluating computer-aided polyp detection systems in realistic temporal settings, would strengthen assessment of clinical readiness. Clinical deployment still depends on full end-to-end latency on the target system, robustness to common artifacts (blood, bubbles, glare, blur, folds), low false positives, and temporally stable overlays. In practice, it should function as an assistive overlay with minimal controls and conservative behavior on low-confidence frames. Clinical use would further require SaMD/AI-CAD evaluation, including multi-center external validation, human-factors testing, cybersecurity and privacy safeguards, audit logging, and a defined model update and change-management plan.

Reference:

[1] Fitting, D., et al. "A video based benchmark data set (ENDOTEST) to evaluate computer-aided polyp detection systems." Scandinavian Journal of Gastroenterology 57.11 (2022): 1397–1403.
